# Work Engagement, Burnout and Personal Accomplishments Among Social Workers: A Comparison Between Those Working in Children and Adults’ Services in England

**DOI:** 10.1007/s10488-018-0872-z

**Published:** 2018-04-26

**Authors:** Shereen Hussein

**Affiliations:** The Policy Institute at King’s, King’s College London, Strand, London, WC2R 2LS UK

**Keywords:** Maslach burnout inventory, Mental wellbeing, Emotional work, Children and families social work, Adult social work

## Abstract

Social workers (SWs) provide emotional and practical support to vulnerable service users who are likely to suffer from emotional trauma and mental health conditions. Stress and burnout levels are reported to be high among SWs, however, little is known about their relationships with different characteristics. The current article utilises unique and large dataset (n = 3786) on SWs working in adults and children’s services to examine factors associated with burnout. Employing job-demand/resources model and structural equations modelling, we highlight the varying significant impact of work-engagement, administrative support and work experience as moderating factors to burnout across adult and children service specialism in this sample.

## Introduction

Social workers (SWs) play a crucial role in maintaining the health and wellbeing of vulnerable children, adults, older people and their carers. They have a direct role in improving the lives of vulnerable individuals with complex social, physical and mental needs. In the UK, following the establishment of the welfare state, SWs initially focused on poverty, mainly reflecting concerns about the problems of children and families. By the 1930s, the new occupation had achieved professional status as a personal service profession, that are interested with the welfare of wider client groups within various settings from the community to specialist hospitals and institutional units. In England, social work has been provided, in the main, by local authorities (LAs) with SWs working in teams either specializing in children and families (CFSWs) or adults and older people (ASWs) services.

SWs support clients who are, in most cases, socially disadvantages and/or exposed to further negative circumstances such as family violence, homelessness and substance misuse (Ford et al. 2007). Through their professional role, SWs usually deal with life long trauma, loss and abuse and other experiences that might be lost in purely medical perspectives. An increasing research base highlights higher levels of stress and burnout among SWs than other human service occupations (Lloyd et al. 2002; Kim and Stoner 2008). The higher level of stress among SWs is conceptualized to be linked to the nature of social work and the role of human agency in delivering support in highly emotional contexts (Mänttäri-van der Kuip 2014). There are various definitions for the state of ‘burnout’, which was first introduced in the 1970s as ‘a reaction to interpersonal stressors on the job’ (Maslach et al. 2001). Current leading authors in this area define burnout further as ‘a syndrome of emotional exhaustion, depersonalization, and reduced personal accomplishment’ (Maslach and Leiter 2008). The causes of burnout and stress among all SWs include inadequate staffing, excessive workload, poor leadership, lack of support, lack of opportunity for skills development and negative public image (Bove and Pervan 2013; Graber et al. 2008).

The more vulnerable and emotionally presented the service users are the more challenging the relationship with the professional SW is, with higher potential of emotional fatigue. This can explain higher prevalence of stress and burnout observed among SWs providing support to children and adults with learning disabilities and mental health needs than SWs working with other client groups (Hussein et al. 2014a, b; Edwards et al. 2010; Evans et al. 2006; Hamama 2012; McFadden et al. 2017). CFSWs could thus be theorized to face additional stressors than other SWs due to concepts of ‘working alliance’ (Bordin 1979) and ‘emotional labour’ where the bond between client and SW can impact on professionals’ wellbeing and stress. Most children receiving social work support are presented in distress and often with experiences of abuse and mistreatment (Bazalgette et al. 2015). Where CFSWs are required to collaborate with often stressed and ‘troubled’ family members with multiple and complex needs who might be presented as challenging or hostile to professional SWs (Morris 2013). Furthermore, some research linked stress among CFSWs to uncertainties and pressures related to their professional role and organizational context that have aroused from fast changing policy context of children social work in England (Hussein 2018; Russ et al. 2009). Additional stressors on CFSWs might relate to societal perception and negative media representations and blame associated with unfortunate outcomes, particular if a child death scandal arose (Cree et al. 2015; Warner 2015). However, SWs working with other clients’ groups are also subjected to negative public image that positions social work as a ‘stigmatized’ occupation, and workers associated with such occupation, where a considerable proportion of clients might suffer from mental health conditions, are found to be more prone to stress (Bove and Pervan 2013; Johnson et al. 2017).

The job-demand/resources (JD–R) model presents a suitable framework to understand and predict SWs burnout and engagement, and consequently organizational performance (Bakker and Demerouti 2007). This model argues that high job demands exhaust employees’ mental and physical resources and therefore lead to the depletion of energy and to health problems. In contrast, job resources, including adequate supervision, foster employees’ engagement and higher sense of personal accomplishments (Locke et al. 2017). The Maslach burnout inventory (MBI) is a widely used measure of burnout as it has the advantage of including both negative items (for exhaustion and depersonalization) and positive items (personal accomplishment) of workers’ wellbeing, and thus captures both sides of that construct (Maslach et al. 1996). The three key dimensions of the MBI are an overwhelming exhaustion (EE: emotional exhaustion), feelings of cynicism and detachment from the job (Dp: depersonalization), and a sense of ineffectiveness and lack of accomplishment (low level of personal accomplishment, PA) (Maslach 1993).

Research has shown that the nature of the task in hand is an important determinant of whether someone experience work engagement, which is directly linked to emotional exhaustion and levels of burnout (Schaufeli and Salanova 2011). While the original JD–R model placed a significant emphasis on how organizations influence the job demand and resources, Bakker and Demerouti (2017) furthered the discussion to highlight the significant role of the individual employee as a proactive agent in interacting with and responding to the organizational design of the job. They have used the term ‘job crafting’, originally coined by Wrzesniewski and Dutton (2001), to describe how individual workers might interact and alter their specific work tasks to make it more meaningful. This highlights the importance of how the individual workers perceive their working tasks and how they amend, or have the ability to amend, such tasks to make their work more meaningful and rewarding. However, the ability of individuals to ‘craft’ their tasks is co-dependent on the flexibility and adaptability of tasks as well as workers’ own autonomy and decision authority.

Research also shows that employees can be highly engaged in difficult and emotionally demanding work, such as that of social work (George 2011). In such situations, workers resort to their social capital including self-efficacy, self-esteem and optimism to manage their emotional exchange with clients (Luthans et al. 2007). Leiter and Maslach (2009) examining nurses’ work experience and burnout, show that such experience could be captures by a continuum of burnout to engagement. They argue that if burnout is at one end then work engagement represents the other end of that continuum. Various factors interact to shape the level of work engagement including workload, involvement in decision-making and equity within the workplace among others. There is little research on measures of work engagement in SW practice with few exceptions (e.g. Hussein et al. 2014b).

In addition to task related and institutional factors, personal characteristics are likely to impact on SWs’ own perception of stressors and engagement (Halbesleben and Buckley 2004). Parker and Griffin (2011) suggest that knowledge and skills, which are products of training and work-experience, may moderate the engagement–stress link and thus should be considered when examining burnout.

High levels of stress, if not managed appropriately, can contribute to burnout and impact on the effectiveness of care delivery to vulnerable people (Skirrow and Hatton 2007) as well as SWs’ own wellbeing and health outcomes (Kim et al. 2011; Johnson et al. 2017). Numerous studies established a link between workers’ stress and various health conditions, most notably cardiovascular disease (Hallqvist et al. 1998; Landsbergis and Theorell 1999); musculoskeletal disorders (Hoogendoorn et al. 2000) and mental health conditions (Nieuwenhuijsen et al. 2010).

Utilising large and unique two datasets that are specific to ASWs and CFSWs in England with comparative information, the current study aims to establish, which work-related and individual aspects are associated with positive or negative outcomes of SWs’ wellbeing as measured by the MBI. Furthermore, it compares the experience of the two groups of SWs in relation to their main client groups (CFSWs and ASWs).

## Data and Methods

### Data

Data used for the current analysis have originally belonged to two national evaluations of social work practices that focused on CFSWs (Hussein et al. 2014a) and on ASWs social workers in England (Manthorpe et al. 2014). The original studies had adopted matched control designs where SWs from both the pilot sites and comparative LAs were included in the studies. As part of the evaluations similar surveys were distributed at two time points for each group of SWs, thus resulting on similar data collected at four overlapping time points. Data were collected from SWs in 22 LAs in England, and survey response rate for each LA ranged between 43 and 60%, which is adequate for this type of surveys. To ensure representativeness and generalizability of the findings, we compared participating samples’ characteristics to aggregate social work profile at each of the LAs. Samples where not statistically different from the population of SWs according to key characteristics such as age and gender at 95% confidence level (see Hussein et al. 2014a; Manthorpe et al. 2014). The original evaluations were funded by The English Department for Education and Department of Health, and the current analysis received separate funding from The English Department of Health.

The survey aimed to capture key organisational and personal characteristics associated with positive work outcomes such as job satisfaction, low level of burnout and low turnover. The survey design was based on qualitative interviews with SWs (a total of 52 interviews: 31 with CFSWs and 21 ASWs) to establish an understanding of which characteristics are likely to be important in predicting various outcomes. The qualitative interviews were analysed thematically and used to derive the survey questions on level of engagement at work and perceptions of various elements of how the work is performed and supported within the organisation (see Hussein et al. 2014a).

### Participants’ Recruitment and Ethical Statement

Practitioners were recruited through their employers who were invited to take part in the research. Employers produced a list of electronic contact details for the research team. Practitioners were contacted directly by the team with a request to complete an electronic survey with the option to opt out from participation. Ethical approval for the original studies were obtained from King’s College London and the Institute of Education’s Research Ethics Committees and from research governance committees in local authorities and further ethical approval for the secondary data analysis presented here was obtained from the author’s institution. As part of the ethical engagement, the research team designed and prepared aggregated, standardized and individualized finding sheets for each of the participating LAs. These were presented to staff and management teams at workshops organized by the team. One of the purposes of this communication method was to counter any potential negative impact of findings related to high levels of burnout in some LAs for example. Alongside the findings, the team also presented research evidence on key factors related to workforce outcomes and quality of work.

### Study Participants

In total, there were 3786 SWs from 22 diverse LAs in England completing similar surveys from 2010 to 2013. Table 1 presents the distribution of participants by all variables included in the analysis.

**Table 1:** Characteristics of social workers participating in the studies by specialism

Characteristics of social workers	Specialism		Total
	Adult 2012–2013	Children 2010–2011	
Personal characteristics			
Gender (**Gen**)			
Female	82.0%	84.3%	82.9%
Male	18.0%	15.7%	17.1%
Self-reported health (**SRH**)			
Fair/good/excellent	94.7%	93.1%	94.1%
Poor/very poor	5.3%	6.9%	5.9%
Ethnicity (**Eth**)			
White	87.4%	84.5%	86.2%
BME	12.6%	15.0%	13.8%
Mean (**Age**)	46.5	44.0	45.5
σ	10.2	10.3	10.3
Work-related characteristics			
Mean number of years working in social care (**Exp1**)	14.5	13.5	14.1
σ	9.5	9.6	9.5
Mean number of years in post (**Exp2**)	5.8	4.4	5.2
σ	5.4	4.4	5.1
Agree/strongly agree: I provide the right level of direct work with service users (**DW1**)	39.4%	24.2%	33.3%
Agree/strongly agree: I provide the right level of direct work with carers (**DW2**)	38.1%	27.3%	33.8%
Agree/strongly agree: I spend the right amount of time completing forms and writing reports (**Form**)	21.1%	15.3%	18.8%
Agree/strongly agree: I spend the right amount of time in meetings and reviews (**Meet**)	50.1%	47.1%	48.9%
Agree/strongly agree: Staff are involved in decision making (**DM**)	61.3%	67.9%	63.9%
Agree/strongly agree: Innovative practice is encouraged (**Inno**)	63.7%	64.0%	63.8%
Agree/strongly agree: Mistakes are treated as opportunities to learn (**Mis**)	65.9%	59.3%	63.3%
Agree/strongly agree: Staff turnover is low (**T-O**)	55.5%	35.6%	47.6%
Agree/strongly agree: I feel confident to challenge practice decisions (**Conf**)	58.0%	59.2%	58.5%
Agree/strongly agree: Staff supervision is a priority where I work (**Sup**)	62.8%	70.8%	66.0%
Total number of cases	2275	1511	3786

Names in bold are used as abbreviation in the models’ diagrams

### Measurement Instruments

All participants completed similar questionnaires inclusive of the MBI and detailed questions on personal and job characteristics. The MBI includes 22-item, 6-point anchored Likert-type scales with three components: “emotional exhaustion” (EE), “depersonalization” (Dp) and “personal accomplishment” (PA) (MBI questions are listed in Box 1).

**Box 1:**
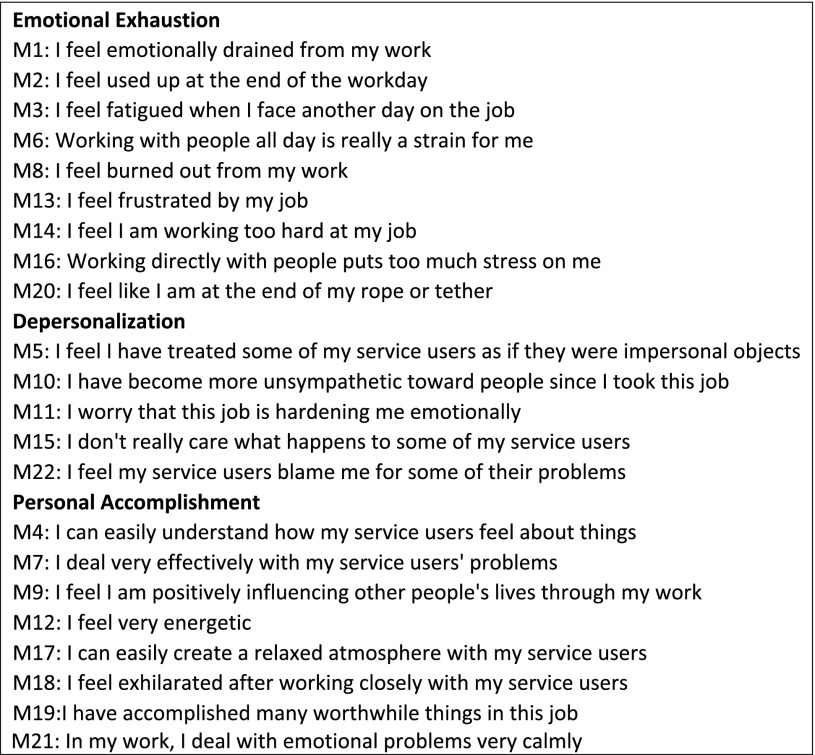
MBI standardised questions (answers from 0 ‘never’ to 6 ‘always’)

In addition to the MBI, the surveys collected similar information on personal and work related characteristics. We ran an exploratory factor analysis model in R to construct latent factors reflecting how the work is organized and delivered based on the set of questions developed during the qualitative phase of this study. The factor analysis identified four work-related factors as following:

- *Work experience* (*Exp*) measured by three items: A1—number of years working in the sector (Exp1); A2—number of years working with the same employer (Exp2); and A3—age.
- *Work engagement* (*Eng*) measured by six items: B1—reported confidence to challenge practice decisions (Conf); B2—ability to adopt innovative practice (Inno); B3—mistakes are used as an opportunity for learning (Mis); B4—involvement in decision making (DM); B5—supervision support is a priority in their work place (Sup); and B6—staff turnover is kept low (T-O).
- *Nature of task* (*DW*) measured by two items: perception of spending the right amount of time in: C1—direct work with clients, whether children or adults (DW1); C2—direct work with their carers (DW2).
- *Resources and support* (*Admin*) measured by two variables: perceptions of spending the right amount of time in: D1—completing forms (Form) and D2—meetings with other professionals (Meet).

Table 2 presents the results of the factor analysis (factor loading) and inter-consistency (measured by Cronbach Alpha) of each of these four factors. The results show that all factors have either acceptable or good level of inter-item consistency. In the context of SW, the latent factor ‘DW’ could be viewed as representing the amount of time spent on preferred tasks (i.e. working directly with clients and their carers as identified during the qualitative analysis of the interviews); while the latent factor ‘Admin’ may present allocated time to less preferred tasks (i.e. administrative work and meetings with other professionals). The correlation matrix between the MBI questions and different variables used to construct these four factors show small cross-item correlation, suggesting that these factors measure different components and latent factors from those measured by the MBI scale (see Table 3).

**Table 2:** Results of factor analysis (factor loading and inter-consistency measure) to identify latent factors representing various work characteristics

Latent factors and their corresponding variables	Component				Cronbach alpha standardised
	Factor 1	Factor 2	Factor 3	Factor 4	
A: Work experience (EXP)					0.71
A1—Exp1: Length of time in years working in social work	0.818	− 0.154	0.157	− 0.020	Good
A2—Exp 2: Length of time in current post	0.689	− 0.133	0.091	0.015	
A3—Age	0.778	− 0.167	0.159	− 0.026	
B: Wok engagement (Eng)					0.671
B1—Conf: Staff feel confident to challenge practice decisions	0.046	0.645	0.351	− 0.067	Acceptable
B2—Inno: Innovative practice encouraged	− 0.038	0.516	0.251	− 0.100	
B3—Mis: Mistakes are treated as opportunities to learn	− 0.034	0.633	0.270	− 0.012	
B4—DM: Staff involved in decision making	− 0.102	0.534	0.278	− 0.013	
B5—Sup: Staff supervision is a priority	0.045	0.525	0.288	0.005	
B6—T-O: Staff turnover is low	0.251	0.452	0.155	0.046	
C: Nature of task (DW)					0.744
C1—DW1: I spend the right amount of direct work with service users	0.209	0.479	− 0.650	− 0.310	Good
C2—DW2: I spend the right amount of direct work with informal carers	0.179	0.465	− 0.616	− 0.416	
D: Resources and support (Admin)					0.601
D1—Form: I spend the right amount of completing forms and writing reports	0.189	0.356	− 0.344	0.550	Acceptable
D2—Meet: I spend the right amount of time in meetings and reviews	0.074	0.345	− 0.330	0.654	

**Table 3:** Correlation between MBI itemised questions and variables representing latent factors reflecting ‘work engagement’; ‘nature of task’ and ‘resources and support’

MBI itemised questions	Variables representing latent factors used in the models									
	B1	B2	B3	B4	B5	B6	C1	C2	D1	D2
M1	− 0.211	− 0.195	− 0.228	− 0.182	− 0.182	− 0.153	− 0.192	− 0.181	0.176	0.132
M2	− 0.195	− 0.194	− 0.201	− 0.173	− 0.178	− 0.178	− 0.200	− 0.192	0.17	0.141
M3	− 0.242	− 0.215	− 0.257	− 0.222	− 0.246	− 0.208	− 0.206	− 0.187	0.156	0.141
M4	− 0.02	− 0.094	− 0.043	− 0.053	− 0.013	− 0.039	− 0.075	− 0.07	0.058	0.006
M5	− 0.101	− 0.115	− 0.085	− 0.141	− 0.1	− 0.081	− 0.067	− 0.066	0.056	0.074
M6	− 0.159	− 0.148	− 0.133	− 0.165	− 0.086	− 0.067	− 0.074	− 0.082	0.081	0.068
M7	0.136	0.102	0.084	0.084	0.098	0.09	0.117	0.117	− 0.086	− 0.084
M8	− 0.230	− 0.212	− 0.234	− 0.2	− 0.23	− 0.18	− 0.185	− 0.159	0.141	0.127
M9	0.195	0.140	0.118	0.161	0.139	0.116	0.162	0.163	− 0.042	− 0.058
M10	− 0.128	− 0.168	− 0.112	− 0.169	− 0.139	− 0.088	− 0.045	− 0.078	0.103	0.082
M11	− 0.143	− 0.171	− 0.149	− 0.155	− 0.191	− 0.069	− 0.067	− 0.092	0.084	0.077
M12	0.193	0.167	0.18	0.178	0.171	0.125	0.149	0.145	− 0.136	− 0.108
M13	− 0.288	− 0.255	− 0.294	− 0.241	− 0.287	− 0.196	− 0.223	− 0.199	0.179	0.129
M14	− 0.159	− 0.119	− 0.193	− 0.123	− 0.132	− 0.138	− 0.144	− 0.157	0.136	0.119
M15	− 0.082	− 0.061	− 0.078	− 0.051	− 0.064	− 0.029	− 0.038	− 0.059	0.009	0.029
M16	− 0.162	− 0.153	− 0.14	− 0.166	− 0.113	− 0.065	− 0.065	− 0.110	0.095	0.055
M17	0.065	0.059	0.059	0.026	0.045	0.048	0.00	0.015	− 0.034	− 0.073
M18	0.092	0.052	0.067	0.080	0.052	0.033	− 0.036	− 0.004	− 0.005	− 0.082
M19	0.138	0.105	0.117	0.100	0.094	0.109	0.077	0.106	− 0.038	− 0.073
M20	− 0.252	− 0.204	− 0.267	− 0.228	− 0.23	− 0.194	− 0.175	− 0.178	0.122	0.124
M21	0.105	0.072	0.065	0.066	0.06	0.056	0.073	0.084	− 0.098	− 0.065
M22	− 0.130	− 0.122	− 0.125	− 0.096	− 0.138	− 0.073	− 0.110	− 0.103	0.14	0.122

### Analysis

The analysis presented here started by investigating the differences between burnout levels among children and adults’ SWs. For exploring the differences in scores of the three elements of burnout (EE, DP and PA) among ASWs (group 1) and CFSWs (group 2), we employed a Bayesian estimation model for two groups’ means, standard deviations and effect size as explained in Kruschke (2013). We implemented this methodology using CmdStan software (Carpenter et al. 2017; Stan Development Team 2017).

As MBI measures burnout through three inter-correlated elements (EE, Dp and PA), with no means of having a summary measure for an overall burnout outcome, structural equations modelling (SEM) was deemed the most appropriate technique to examine the relationship between various work and personal characteristics on the three elements of burnout simultaneously. Two levels of analysis were conducted. First, descriptive and principal component analysis, to establish specific work-related factors, were conducted using R statistical environment (R Core Team 2017); then SEM was conducted using MPlus ver. 7 (Muthén and Muthén 1998–2011).

Comparative analyses of CFSWs and ASWs, reported in the “Findings” section, as well as previous research indicate variable levels of burnout among SWs supporting the two service groups. Furthermore, the two groups reported different levels of satisfaction and engagement with various work related elements (see Table 1). We thus theorized that SWs working with each of the two service groups experience different levels of emotional demand, where personal and related factors may have different implications on each of two groups. To acknowledge these differences, we conducted two separate models, one for each group, to capture these relationships more accurately. The models employed the identified four latent factors as confirmatory factor analysis (CFA) within their measurement models. Theses models examined the relationships between EE, Dp and PA and the identified four latent factors as well as other measured personal characteristics such as gender (Gen), self-reported health (SRH) and ethnicity (Eth).

Bayesian analysis is establishing a position in organisational studies as a more attuned method than frequentist statistics. It is argued that traditional analyses using maximum likelihood (ML) and likelihood-ratio χ^2^ testing apply unnecessarily strict models to represent hypotheses derived from substantive theory, often leading to rejection of the model (Zyphur and Oswald 2015). In contrast, Bayesian analysis does not rely on large-sample theory and provides the whole distribution of predicted posterior probability not assuming that it follows the normal distribution. We conducted two Bayesian SEM models: model 1 used data obtained from ASWs (n = 1998) and model 2 uses data from CFSWs (n = 1316), after list-wise deletion of missing values.

### Fit Indices

ML estimation methods were used and the input for each analysis was the covariance matrix of the items. The goodness-of-fit of the models were evaluated using the χ^2^ goodness-of-fit statistic (Hoyle 1995). Model 1 (ASWs) goodness of fit was acceptable at *p* = 0.487; and the Bayesian posterior predictive checking indicated that the 95% credible intervals for the difference between observed and replicated χ^2^ was (− 29.095, 31.074). The corresponding statistics for model 2 (CFSW) were *p* = 0.508; and 95% CI of (− 30.067, 31.955).

## Findings

### Variations in Burnout Levels Between CFSWs and ASWs

Table 1 indicates no significant differences between CFSW and ASWs according to gender and self-assessed health, while CFSWs were slightly more ethnically diverse (χ^2^ = 5.74; *p* = 0.017); and were on average slightly younger (*F* = 57.2, *p* < 0.001) than adults’ SWs. The latter differences are likely to be related to the higher contribution of migrant workers in CFSW, this group are characterised in general by younger age and are ethnically diverse (Hussein 2014). On the other hand, Table 1 shows that ASWs tended to have significantly more positive views about their levels of work engagement and were more experienced with higher mean number of years in the sector as well as in their current posts (*F* = 8.9 and 75.1; *p* = 0.003 and *p* < 0.001 respectively).

Using Maslach et al. (1996) standard grouping, on average, all SWs included in this study (both CFSWs and ASWs), had moderate EE (µ = 22.3; σ = 10.4); borderline low Dp (µ = 5.9; σ = 4.3) and borderline moderate PA scores (µ = 31.9; σ = 6.1).

**Fig. 1:**
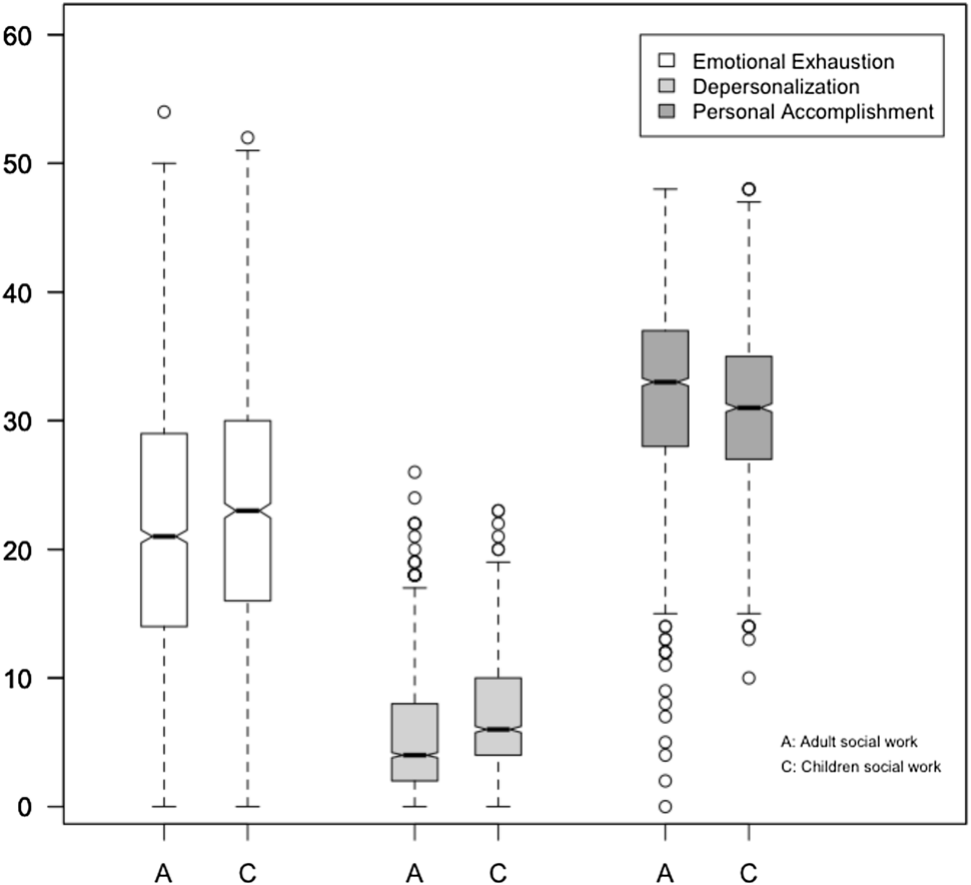
Burnout levels, measured by MBI by social work specialism

Estimates of the effect size of working with children or adults and their credible intervals are summarized in Table 4. The findings presented in Table 4 show significant differences according to main service group, with CFSWs scoring worse than ASWs in all elements of burnout. Figure 1 shows that CFSWs have significantly higher average scores of EE (µ = 23.1 vs. 21.8; *t* = 3.79; *p* < 0.001) and Dp (µ = 6.8 vs. 5.1; *t* = 11.78; *p* < 0.001) and lower PA scores (µ = 31.1 vs. 32.5 *t* = − 6.44; *p* < 0.001) than ASWs. Furthermore, Table 4 shows that the highest effect size of client service group is observed in relation to Dp at 0.30 compared to − 0.17 for PA and 0.09 for EE. These scores mean that CFSWs in general had moderate levels of EE and Dp and low levels of PA, while ASWs displayed moderate levels of EE and PA and low level of Dp (Maslach et al. 1996).

**Table 4:** Estimates of the effect size of service group (1. adults vs. 2. children) on MBI sub-scales

Parameter	Mean	Standard deviation	Credible intervals		
			2.50%	50%	97.50%
Emotional exhaustion					
µ_1_	21.75	0.22	21.31	21.75	22.19
µ_2_	23.05	0.26	22.54	23.04	23.55
σ_1_	10.54	0.16	10.24	10.54	10.86
σ_2_	9.87	0.18	9.52	9.87	10.24
Effect size	0.09	0.02	0.04	0.09	0.13
Depersonalization					
µ_1_	5.10	0.10	4.91	5.10	5.29
µ_2_	6.82	0.11	6.59	6.82	7.04
σ_1_	4.04	0.08	3.87	4.04	4.2
σ_2_	4.16	0.09	3.99	4.16	4.34
Effect size	0.30	0.03	0.25	0.30	0.35
Personal accomplishment					
µ_1_	32.5	0.13	32.24	32.5	32.76
µ_2_	31.09	0.15	30.8	31.09	31.39
σ_1_	5.95	0.12	5.72	5.95	6.19
σ_2_	5.49	0.12	5.25	5.49	5.73
Effect size	− 0.17	0.02	− 0.21	− 0.17	− 0.12

### Factors Influencing ASWs’ Burnout Levels

**Fig. 2:**
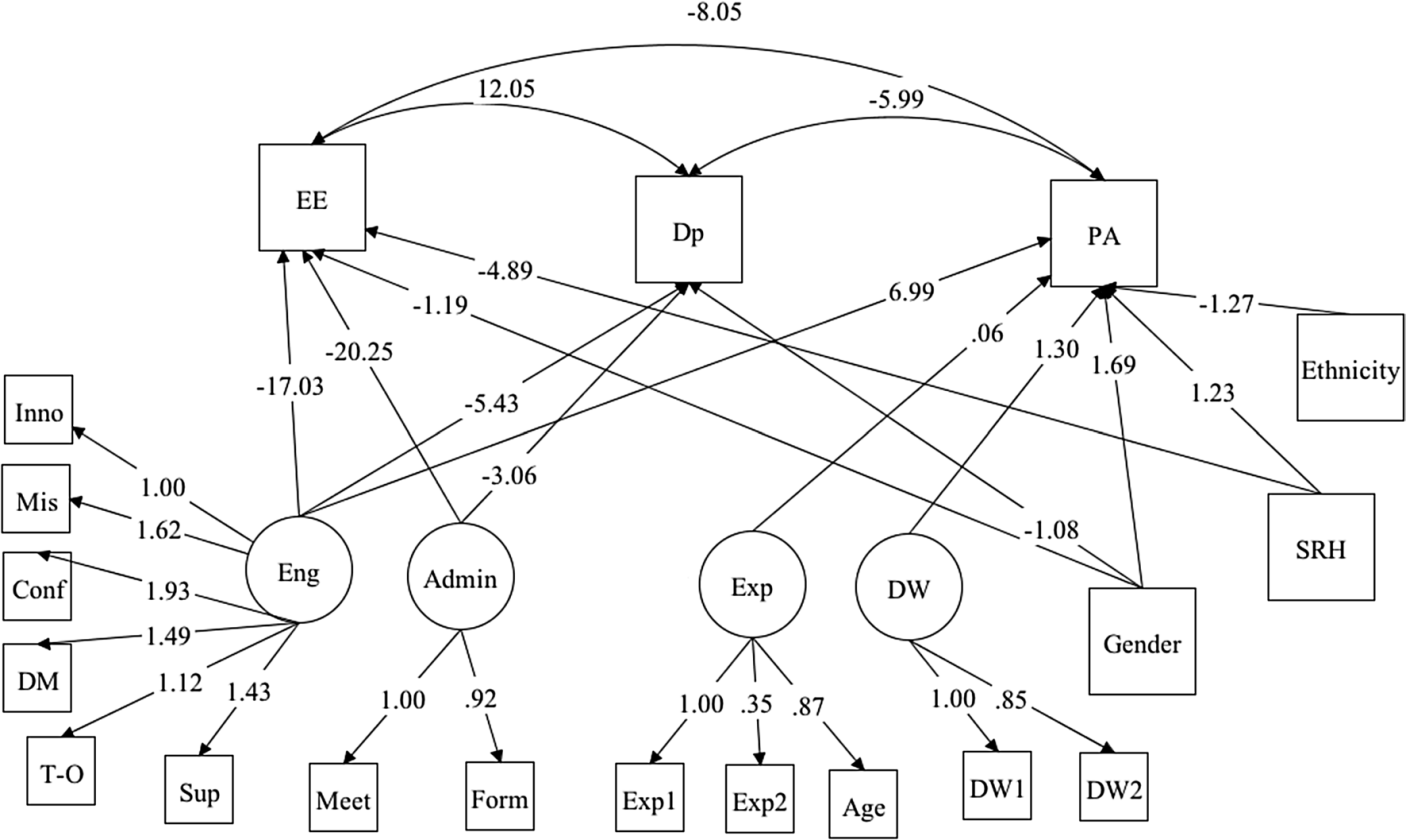
Results of adults’ social workers burnout structural equation model with latent factors, significant associations only

Figure 2 provides visual representations of the significant results of the final SEM model for ASWs’ EE, Dp and PA. Full results of the model are presented in the first set of columns (model 1) in Table 5. The results of the SEM model for ASWs, confirm the theory that EE was positively associated with DP, while negatively associated with PA. Similarly DP is negatively associated with PA. The positive relationship between EE and Dp was the largest in magnitude with (β = 12.05, *p* < 0.001), while PA was negatively associated with both EE and DP (β = − 8.05 and − 5.99; respectively at *p* < 0.001).

**Table 5:** Results of structural equation models for adult and children social workers

SEM with latent factors Significant results only	Adults social workers						Children social workers					
	Posterior			95% CI			Posterior			95% CI		
	Estimate	SD	*p* Value	Lower 2.5%	Upper 2.5%	Sig.	Estimate	SD	*p* Value	Lower 2.5%	Upper 2.5%	Sig.
EE												
ENG	− 17.03	2.44	< 0.001	− 22.02	− 12.35	*	− 8.38	1.23	< 0.001	− 10.80	− 6.00	*
ADMIN	− 20.25	3.50	< 0.001	− 28.58	− 14.68	*	− 16.59	2.34	< 0.001	− 21.83	− 12.64	*
SRH (good/v. good vs. not)	− 4.89	0.91	< 0.001	− 6.67	− 3.10	*	− 4.67	0.97	< 0.001	− 6.57	− 2.80	*
Gender (female vs. male)	− 1.19	0.55	0.014	− 2.27	− 0.12	*	− 0.46	0.68	0.250	− 1.77	0.89	
Dp												
ENG	− 5.43	0.88	< 0.001	− 7.25	− 3.80	*	− 2.95	0.53	< 0.001	− 3.99	− 1.92	*
EXP	− 0.01	0.02	0.196	− 0.04	0.02		− 0.10	0.02	< 0.001	− 0.13	− 0.06	*
ADMIN	− 3.07	1.06	0.001	− 5.32	− 1.19	*	− 3.59	0.84	< 0.001	− 5.33	− 2.03	*
Ethnicity (white vs. BME)	0.40	0.28	0.077	− 0.15	0.94		1.08	0.32	0.001	0.45	1.71	*
Gender (female vs. male)	− 1.08	0.23	< 0.001	− 1.54	− 0.63	*	− 0.66	0.31	0.017	− 1.27	− 0.05	*
PA												
ENG	6.99	1.27	< 0.001	4.64	9.61	*	3.03	0.69	< 0.001	1.72	4.41	*
EXP	0.06	0.02	0.002	0.02	0.11	*	0.10	0.02	< 0.001	0.06	0.15	*
DW	1.30	0.58	0.011	0.21	2.44	*	1.09	0.62	0.037	− 0.11	2.35	
ADMIN	2.83	1.55	0.032	− 0.15	5.97		2.90	1.09	0.002	0.91	5.18	*
Ethnicity (white vs. BME)	− 1.27	0.42	0.002	− 2.08	− 0.44	*	− 1.38	0.43	0.001	− 2.22	− 0.54	*
SRH (good/v. good vs. not)	1.23	0.59	0.018	0.08	2.39	*	1.51	0.62	0.006	0.32	2.71	*
Gender (female vs. male)	1.69	0.36	< 0.001	1.00	2.39	*	0.22	0.42	0.306	− 0.62	1.04	

The results indicate that some factors and variables are significantly associated with only one of the burnout outcomes, while others are associated with two or all of the MBI burnout elements. For ASWs, levels of work engagements (Eng) and administrative support (Admin) had the most significant effects on EE and Dp. Reported better engagement with work significantly reduced EE and Dp (β = − 17.03 and − 5.43 respectively at *p* < 0.001) and perceptions of not spending excessive amount of time in completing forms and meetings with other professionals (less preferred tasks) also reduced the levels of EE and Dp among ASWs (β = − 20.25 and − 3.06; *p* < 0.001 and 0.001 respectively). Levels of PA were significantly and positively associated with ASWs’ own work experience (Exp) as well as the nature of work being direct work with clients and their carers (DW) (β = 0.06 and 1.30; *p* = 0.002 and 0.011 respectively). However, the largest magnitude is observed between PA and ASWs’ work engagement (β = 6.99, *p* < 0.001).

Personal characteristics appeared to have some significant associations with various elements of ASWs’ burnout. Women ASWs displayed significantly lower levels of EE and Dp and higher levels of PA than men (β = − 1.19, − 1.08 and 1.69; *p* = 0.014, < 0.001 and < 0.001 respectively). ASWs’ ethnicity was associated with PA only, where ASWs with white ethnicity displayed significantly lower levels of PA (β = − 1.27, *p* = 0.002). The model indicated that SRH had a strong negative association with EE and positive association with PA; the better the reported health the lower levels of EE and higher levels of PA (β = − 4.89 and 1.23; *p* < 0.001 and 0.018 respectively).

### Factors Influencing CFSWs’ Burnout Levels

**Fig. 3:**
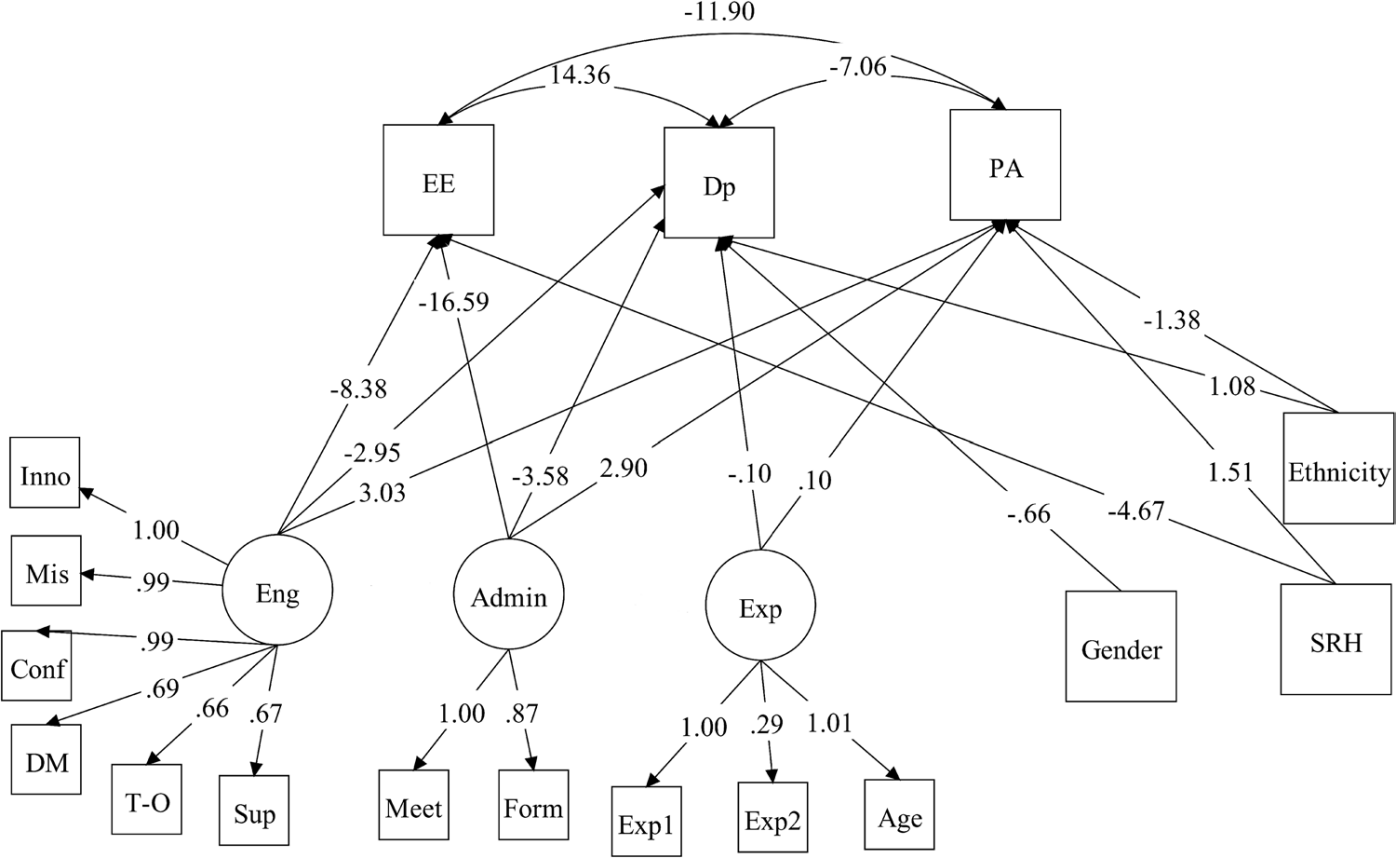
Results of children’s social workers burnout structural equation model, significant associations only

Figure 3 provides visual representations of significant results of the final SEM model for EE, DP and PA among CFSWs. Full results of the model are presented in Table 5, second set of columns (model 2). For CFSWs levels of EE were positively associated with Dp and negatively associated with PA. As levels of burnout were significantly higher among CFSWs than ASWs, the magnitude of the positive association between EE and Dp was also larger among CFSWs than ASWs (β = 14.36, *p* < 0.001).

Similar to ASWs, levels of work engagements (Eng) and administrative support (Admin) had the largest magnitude of association with all burnout outcomes among CFSWs. Higher levels of work engagement were significantly associated with reduced levels of EE and Dp and increased levels of PA (β = − 8.38, − 2.95 and 3.03; *p* < 0.001 respectively). Similarly, spending the right amount of time in administrative tasks was significantly associated with reduced levels of EE and DP and increased levels of PA among CFSWs (β = − 16.59, − 3.58 and 2.90; *p* < 0.001 respectively). It is interesting to note that, unlike ASWs, the nature of tasks being direct work with children and their carers was not significantly associated with any of the burnout outcomes among CFSWs. How experienced CFSWs was significantly associated with both DP and PA; with more experienced staff displaying significantly lower levels of Dp and higher levels of PA (β = − 0.10 and 0.10; *p* < 0.001 respectively).

In relation to personal characteristics, women CFSWs had significantly lower Dp scores than men (β = − 0.66; *p* < 0.001). Unlike ASWs, for CFSWs, gender did not appear to be significantly associated with other elements of burnout. On the other hand, ethnicity was associated with both Dp and PA, where CFSWs with white ethnicity displaying significantly higher levels of Dp and lower levels of PA than workers from black and minority ethnic (BME) (β = 1.08 and − 1.38; *p* = 0.001 respectively). Better SRH was significantly associated with lower levels of EE and higher levels of PA among CFSWs (β = − 4.67 and 1.51; *p* < 0.001 and 0.006 respectively).

## Discussion

SWs’ mental wellbeing and feelings of personal accomplishments influence their ability to meet the needs of vulnerable service users, many of whom suffer from complex social and mental health needs. Working with particular client groups and with various levels of work-engagement and autonomy impact SWs’ positive and negative feelings in relation to their job. Rarely available comparative data on SWs in England offer a unique opportunity to consider potential impact of the client group on SWs’ burnout and work engagement levels. The data relate to SWs employed by LAs and thus capture those professionals who have statuary duties to meet service users’ social and mental health needs.

The findings highlight that ASWs generally report higher levels of work engagement; satisfaction with time allocated to direct work with clients and have longer years of work experience in the sector and in their posts than CFSWs. It is important to note that the largest effect size of the client group on burnout is observed in relation to Dp, where CFSWs had higher levels of Dp than ASWs. These differences reflect some of the implicit effects of the client group, where ‘work alliance’ with clients and the level of emotional labour among CFSWs could be theorized to be higher than that observed among ASWs. CFSWs work in the main with what is coined in the UK as ‘troubled families’ or ‘families at risk’, where most family members suffer from multiple and complex social problems that require intensive intervention from CFSWs and other professionals (Morris 2013). In such situations, CFSWs attempt to work with stressed and often hostile family members to deliver difficult professional advice (Ferguson 2011). Furthermore, the widespread negative media attention when young children are portrayed to suffer while in the care of professional SWs exacerbates negative feelings among CFSWs (Cree et al. 2015; Warner 2015). Other factors such as the chronic high turnover rates traditionally observed among CFSWs in England (Department for Education 2017) might also explain the higher burnout rates observed among CFSWs in the current study.

Overall levels of burnout observed among SWs in this study could be regarded as moderate to high according to Maslach standardized grouping (Maslach et al. 1996), with CFSWs significantly displaying higher levels of emotional exhaustion and depersonalization and lower levels of personal accomplishment than ASWs. The structural equation modeling adopted for this study, highlights a number of important factors contributing to experiencing burnout among the two groups of SWs, these are categorized within the JD–R model. The findings illustrate that SWs’ burnout is determined by a range of work-related factors that can be categorized as either job-demands or job-resources. Job resources are identified in the literature to be manifested through the levels of work engagement: for example, when SWs perceive their organization to provide a supportive and involving climate, where they can contribute to important decisions and take ownership of their work (Leiter and Maslach 2009; Schaufeli and Salanova 2011). This body of research indicates that the more engaged the workers are the more likely they have higher levels of PA and lower levels of EE and Dp (Mackie et al. 2001). The results presented in this study resonate with this body of literature as levels of work-engagement had one of the highest magnitudes of effect for both ASWs and CFSWs in relation to burnout, particularly in minimizing levels of EE. The current study highlights that measuring work-engagement among SWs is complex and has various dimensions to it. In this study, work-engagement was captured through several self-perceived factors, such as SWs confidence to challenge practice decisions, their ability to adopt innovative practice, a culture of accepting mistakes as opportunities for learning, being involved in decision making, feelings that supervision and support are priorities and a general perception of low staff turnover.

The findings show that perceived level of resources and support with administrative tasks had a large impact on reducing EE levels among SWs regardless of their client group, and for ASWs it also enhanced their levels of PA. Having practical support with paper work and administrative tasks, while seems a simple target to achieve, has considerable impact on SWs wellbeing.

The nature of tasks and time allocated to achieve them can be quite important in determining human services workers’ general wellbeing as well as levels of job satisfaction and intention to quit (George 2011; Schaufeli and Salanova 2011). The qualitative phase of this study highlighted that SWs preferred be more engaged working directly with clients, as this is often cited as their main motivation to choose this career (Stevens et al. 2012), while they can be less engaged completing administrative tasks and filling forms as they don’t attach the same value and reward to such tasks (Hussein et al. 2014a, b; McFadden et al. 2015). The SEMs results show that the perceived right amount of direct work with clients and their carers significantly and positively impacts the levels of PA for ASWs but has no significant relationship with CFSWs’ burnout measures. It is possible that when controlling for other factors in our models, such as work-engagement and administrative support, which are more important in relation to predicting burnout levels among CFSWs, the impact of direct work becomes less significant.

Parker and Griffin (2011) argue that individuals with a good understanding of the broader goals of their occupation and organization are likely to direct their efforts appropriately with positive implications on both their wellbeing and work outcome. Work experience might also relate to the ability and autonomy of workers to ‘craft’ their tasks in a positive way (Bakker and Demerouti 2017). The current analysis considers the impact of the level of work-experience of SWs within the whole sector and within their particular organisation on their levels of burnout. The findings indicate that ‘work-experience’ has a significant role to play in improving SWs’ burnout levels. This was particularly observed among CFSWs, where work-experience is associated with lower levels of Dp and higher levels of PA, while for ASWs the relationship is confined to PA (with a smaller magnitude). This finding is particularly important in the context of higher turnover rates observed among CFSWs in England and calls for improved retention strategies for this group of SWs.

Individual factors refer to individual differences or personal characteristics that are relatively stable over situations and time. Although current evidence indicates the possibility that various aspects of the work environment are more important predictors of burnout than personal characteristics, researchers are advised to consider variations in burnout that are related to personal characteristics (Halbesleben and Buckley 2004). It is likely that cultural and social capital of individual SWs play a role in their perception of, and potentially mitigates, the impact of work-stressors. For example, previous research indicates that SWs with personal experience of mistreatment have higher risk of experiencing secondary trauma when faced with similar situations in their professional lives (McFadden et al. 2015). While the current data did not allow the inclusion of indicators of workers’ life histories and potential experience of trauma; they enabled accounting for key personal characteristics, such as health, gender and ethnicity.

The findings point to the important role of gender in the experience of various elements of burnout, with women CFSWs displaying significantly lower levels of Dp, while women ASWs performing significantly better in all measures of burnout than men. These findings are, to some extent, comparable to previous research where men in different occupations tend to have higher Dp levels (Purvanova and Muros 2010). It is likely that such differences correlate with the perception that social work, in general, could be regarded as a ‘female-dominated’ occupation. However, it also might be a result of confounding effects of other factors not measured in this study, such as social support at home and specific cultures/social capital. The direction of association between ethnicity and EE and PA is interesting, where white British CFSW display higher levels of EE and white British SWs display lower levels of PA. Similar findings are observed in previous research focusing on hospital and community-based mental health workers in the UK (Prosser et al. 1996; Edwards et al. 2010). This could be conceptually linked to either a higher degree of association between British workers and service users or to different levels of social support from kin and informal networks between white British and BME workers. It was not possible to capture these differences using the current data.

The relationship between burnout and health is two directional in nature. Vast research evidence shows a significant relationship between burnout and physical and mental health (Hoogendoorn et al. 2000; Landsbergis and Theorell 1999; Nieuwenhuijsen et al. 2010). On the other hand, poor health could be the cause of burnout, for example, it might be more difficult for workers with poorer health to manage their workload and to transform their work engagement into higher levels of personal accomplishments (McFadden et al. 2015). The current analysis highlights the strong relationship between SRH and levels of EE and PA among both ASWs and CFSWs but it could not establish the direction of such relationship.

### Limitations

There are a number of limitations to this study that should be acknowledged. First, there is a time difference between the CFSWs and ASWs surveys, during this period of time, the broader English social work policy has seen some developments, which might have impacted on the experience of SWs, however, these developments have a lagged effect and are unlikely to impact the overall burnout levels immediately. The current data did not collect information on SWs own experience of traumatic experience nor on the social support they receive outside of the workplace, having such information would have been useful to understand the bi-directional relationship between home and work stress. A longitudinal approach in data collection and analysis would enable establishing the direction of the relationship between health and various elements of burnout.

## Conclusion

The current study confirms the important role of work-engagement and resources as mitigating factors for burnout among SWs regardless of their main service group. The relatively easy goal of ensuring support with administrative tasks appears to have considerable influence on SWs experience of burnout and thus should be promoted within social work settings. Work experience reflecting greater awareness of the structure and goals of the sector and specific organisational structures are more important in mitigating the impact of burnout among CFSWs than ASWs. This calls for taking appropriate measures for improving retention and reducing turnover rates among CFSWs. Personal characteristics, such as gender and ethnicity, are significantly associated with various levels of burnout and personal accomplishments. Further research into the exact dynamics of these characteristics is needed to inform both theory and organizational policies.

## References

[CR1] Bakker AB, Demerouti E (2007). The job demands-resources model: State of the art. Journal of Managerial Psychology.

[CR2] Bakker AB, Demerouti E (2017). Job demands-resources theory: Taking stock and looking forward. Journal of Occupational Health Psychology.

[CR3] Bazalgette L, Rahilly T, Trevelyan G (2015). Achieving emotional wellbeing for looked after children: A whole system approach.

[CR4] Bordin ES (1979). The generalizability of the psychoanalytic concept of the working alliance. Psychotherapy: Theory, Research & Practice.

[CR5] Bove LL, Pervan SJ (2013). Stigmatized labour: An overlooked service worker’s stress. Australasian Marketing Journal.

[CR7] Carpenter, B., Gelman, A., Hoffman, M. D., Lee, D., Goodrich, B., Betancourt, M., et al. (2017). Stan: A probabilistic programming language. *Journal of Statistical Software, 76*(1). 10.18637/jss.v076.i01 PMC9788645 36568334

[CR8] Cree V, Clapton G, Smith M (2015). Revisiting moral panics.

[CR9] Department for Education (2017). Experimental statistics: Children and family social work workforce in England, year ending 30 September 2016.

[CR10] Edwards D, Burnard P, Coyle D, Fothergill A, Hannigan B (2010). Stress and burnout in community mental health nursing: A review of the literature. Journal of Psychiatric Mental Health Nursing.

[CR11] Evans S, Huxley P, Gately C, Webber M, Mears A, Pajak S (2006). Mental health, burnout and job satisfaction among mental health social workers in England and Wales. British Journal of Psychiatry.

[CR12] Ferguson H (2011). Child protection practice.

[CR13] Ford T, Vostanis P, Meltzer H, Goodman R (2007). Psychiatric disorder among British children looked after by local authorities: Comparison with children living in private households. The British Journal of Psychiatry.

[CR14] George JM (2011). The wider context, costs, and benefits of work engagement. European Journal of Work and Organizational Psychology.

[CR15] Graber JE, Huang ES, Drum ML, Chin MH, Walters AE, Heuer L (2008). Predicting changes in staff morale and burnout at community health centers participating in the health disparities collaboratives. Health Services Research.

[CR16] Halbesleben JRB, Buckley MR (2004). Burnout in organizational life. Journal of Management.

[CR17] Hallqvist J, Diderichsen F, Theorell T, Reuterwall C, Ahlbom A, SHEEP Study group (1998). Is the effect of job strain on myocardial infarction risk due to interaction between high psychosocial demands and low decision latitude? Results from Stockholm heart epidemiology program (SHEEP). Social Science Medicine.

[CR18] Hamama L (2012). Differences between children’s social workers and adults’ social workers on sense of burnout, work conditions and organisational social support. British Journal of Social Work.

[CR19] Hoogendoorn W, van Poppel M, Bongers P, Koes B, Bouter L (2000). Systematic review of psychosocial factors at work and private life as risk factors for back pain. Spine.

[CR20] Hoyle RH, Hoyle RH (1995). The structural equation modeling approach: Basic concepts and fundamental issues. Structural equation modeling, concepts, issues, and applications.

[CR21] Hussein S (2014). Hierarchical challenges to transnational social workers’ mobility: The United Kingdom as a destination within an expanding European Union. British Journal of Social Work.

[CR22] Hussein S, Beddoe L, Bartley A (2018). In search of better opportunity: Transnational social workers in the United Kingdom navigating the maze of global and social mobility. Transnational social work: Opportunities and challenges of a global profession.

[CR23] Hussein S, Manthorpe J, Ridley J, Austerberry H, Ferrelly N, Larkins C (2014). Independent children’s social work practice pilots: Evaluating practitioners’ job control and burnout. Research on Social Work Practice.

[CR24] Hussein S, Moriarty J, Stevens M, Sharpe E, Manthorpe J (2014). Organisational factors, job satisfaction and intention to leave among newly qualified social workers in England. Social Work Education, An International Journal.

[CR25] Johnson J, Hall LH, Berzins K, Baker J, Melling K, Thompson C (2017). Mental healthcare staff well-being and burnout: A narrative review of trends, causes, implications, and recommendations for future interventions. International Journal of Mental Health Nursing.

[CR26] Kim H, Ji J, Kao D (2011). Burnout and physical health among social workers: A three-year longitudinal study. Social Work.

[CR27] Kim H, Stoner M (2008). Burnout and turnover intention among social workers: Effects of role stress, job autonomy and social support. Administration in Social Work.

[CR28] Kruschke J (2013). Bayesian estimation supersedes the *t* test. Journal of Experimental Psychology.

[CR29] Landsbergis P, Theorell T (1999). Measurement of psychosocial workplace exposure variables. Self-report questionnaires. Occupational Medicine.

[CR30] Leiter M, Maslach C (2009). Nurse turnover: The mediating role of burnout. Journal of Nursing Management.

[CR31] Lloyd C, King R, Chenoweth L (2002). Social work, stress and burnout: A review. Journal of Mental Health.

[CR32] Locke J, Violante S, Pullmann MD, Kerns SEU, Jungbluth N, Dorsey S (2017). Agreement and discrepancy between supervisor and clinician alliance: Associations with clinicians’ perceptions of psychological climate and emotional exhaustion. Administration and Policy in Mental Health and Mental Health Services Research.

[CR33] Luthans F, Youssef CM, Avolio BJ (2007). Psychological capital: Developing the human competitive edge.

[CR34] Mackie KS, Holahan CK, Gottlieb NH (2001). Employee involvement management practices, work stress, and depression in employees of a human services residential care facility. Human Relations.

[CR35] Manthorpe, J., Harris, J., Hussein, S., Cornes, M., & Moriarty, J. (2014). *Evaluation of the social work practices with adults pilots*. Final Report to the Department of Health. London: King’s College London.

[CR36] Mänttäri-van der Kuip M (2014). The deteriorating work-related well-being among statutory social workers in a rigorous economic context. European Journal of Social Work.

[CR37] Maslach C, Schaufeli WB, Maslach C, Marek T (1993). Burnout: A multidimensional perspective. Professional burnout: Recent developments in theory and research.

[CR38] Maslach C, Jackson S, Leiter M (1996). Maslach burnout inventory manual.

[CR39] Maslach C, Leiter MP (2008). Early predictors of job burnout and engagement. Journal of Applied Psychology.

[CR40] Maslach C, Schaufeli WB, Leiter MP (2001). Job burnout. Annual Review of Psychology.

[CR41] McFadden P, Campbell A, Taylor B (2015). Resilience and burnout in child protection social work: Individual and organizational themes from a systematic literature review. British Journal of Social Work.

[CR42] McFadden P, Manthorpe G, Mallett J (2017). Commonalities and differences in social work with learning disability and child protection: Findings from a UK ‘Burnout’ national survey. British Journal of Social Work.

[CR43] Morris K (2013). Troubled families: Vulnerable families’ experiences of multiple service users. Children & Family Social Work.

[CR44] Muthén, L. K., & Muthén, B. O. (1998–2011). *Mplus user’s guide* (7th ed.). Los Angeles: Muthén & Muthén.

[CR45] Nieuwenhuijsen K, Bruinvels D, Frings-Dresen M (2010). Psychosocial work environment and stress-related disorders, a systematic review. Occupational Medicine.

[CR46] Parker S, Griffin M (2011). Understanding active psychological states: Embedding engagement in a wider nomological net and closer attention to performance. European Journal of Work and Organizational Psychology.

[CR47] Prosser D, Johnson S, Kuipersg E, Szmukler E, Bebbingtonand P, Thornicroft G (1996). Mental health, ‘burnout’ and job satisfaction among hospital and community-based mental health staff. British Journal of Psychiatry.

[CR48] Purvanova R, Muros J (2010). Gender differences in burnout: A meta-analysis. Journal of Vocational Behavior.

[CR49] R Core Team (2017). R: A language and environment for statistical computing.

[CR50] Russ E, Lonne B, Darlington Y (2009). Using resilience to reconceptualise child protection workforce capacity. Australian Social Work.

[CR51] Schaufeli WB, Salanova M (2011). Work engagement: On how to better catch a slippery concept. European Journal of Work and Organizational Psychology.

[CR52] Skirrow P, Hatton C (2007). ‘Burnout’ amongst direct care workers in services for adults with intellectual disabilities: A systematic review of research findings and initial normative data. Journal of Applied Research in Intellectual Disabilities.

[CR53] Stan Development Team. (2017). CmdStan: The command-line interface to Stan, Version 2.17.0. http://mc-stan.org.

[CR54] Stevens M, Sharpe E, Manthorpe J, Moriarty J, Hussein S, Orme J (2012). Helping others or a rewarding career? Investigating student motivations to train as social workers in England. Journal of Social Work.

[CR55] Warner J (2015). The emotional politics of social work and child protection.

[CR56] Wrzesniewski A, Dutton JE (2001). Crafting a job: Revisioning employees as active crafters of their work. The Academy of Management Review.

[CR57] Zyphur, Oswald F (2015). Bayesian estimation and inference: A user’s guide. Journal of Management.

